# Characterization of two β-galactosidases LacZ and WspA1 from *Nostoc flagelliforme* with focus on the latter’s central active region

**DOI:** 10.1038/s41598-021-97929-6

**Published:** 2021-09-16

**Authors:** Xiang Gao, Litao Liu, Lijuan Cui, Tao Zheng, Boyang Ji, Ke Liu

**Affiliations:** 1 School of Food and Biological Engineering, Shaanxi University of Science and Technology, Xi’an, 710021 China; 2 School of Life Sciences, Central China Normal University, Wuhan, 430079 China; 3 Department of Biology and Biological Engineering, Chalmers University of Technology, 41296 Gothenburg, Sweden

**Keywords:** Biochemistry, Biotechnology

## Abstract

The identification and characterization of new β-galactosidases will provide diverse candidate enzymes for use in food processing industry. In this study, two β-galactosidases, Nf-LacZ and WspA1, from the terrestrial cyanobacterium *Nostoc flagelliforme* were heterologously expressed in *Escherichia coli*, followed by purification and biochemical characterization. Nf-LacZ was characterized to have an optimum activity at 40 °C and pH 6.5, different from that (45 °C and pH 8.0) of WspA1. Two enzymes had a similar Michaelis constant (*Km* = 0.5 mmol/liter) against the substrate o-nitrophenyl-β-D-galactopyranoside. Their activities could be inhibited by galactostatin bisulfite, with IC50 values of 0.59 µM for Nf-LacZ and 1.18 µM for WspA1, respectively. Gel filtration analysis suggested that the active form of WspA1 was a dimer, while Nf-LacZ was functional as a larger multimer. WspA1 was further characterized by the truncation test, and its minimum central region was found to be from residues 188 to 301, having both the glycosyl hydrolytic and transgalactosylation activities. Finally, transgenic analysis with the GFP reporter protein found that the N-terminus of WspA1 (35 aa) might play a special role in the export of WspA1 from cells. In summary, this study characterized two cyanobacterial β-galactosidases for potential applications in food industry.

## Introduction

β-galactosidase (EC 3.2.1.23), commonly known as lactase, is one of the most important enzymes used in food processing industry^1,2^. This enzyme catalyzes the hydrolysis of β-galactosides from polymers, oligosaccharides or secondary metabolites by breaking the β-D-galactosidic linkages^3^. In addition to the hydrolytic activity, some β-galactosidases also possess the transgalactosylation activity, which involves the process of transfering galactose to another carbohydrate instead of water^4^. Thus, this enzyme has two main applications including the removal of lactose from milk products and the production of galactosylated products such as galacto-oligosaccharides (GOS). The lactose-hydrolyzed milk products can meet the need of lactose-intolerant people, while GOS is one of the important human prebiotics^5^.

β-galactosidases are distributed in a variety of sources including bacteria, fungi, and plants^1,6^. They are categorized into the glycoside hydrolase (GH) families GH1, GH2, GH35, GH42, GH59, and GH147 based on their similarities^2^. β-galactosidases from these GH families belong to the superfamily Clan-A and share the (α/β)8 barrel structure^7^. The well-known *Escherichia coli* β-galactosidase LacZ belongs to the GH2 family and has been structurally elucidated^8,9^. The functionally active form of *E. coli* LacZ is a homotetramer with each monomer comprising of five structural domains, and the third (central) domain (residues 334–627) is an (α/β)8 barrel with an extended active-site cleft. Different sources of β-galactosidases differ in their optimum pH and temperature, thermal stability, substrate specificity, and metal ion cofactor sensitivity, providing a diversified selection for application in food processing^1,2,10–12^. Therefore, identification and characterization of new β-galactosidases from natural resources is beneficial for establishing glycosidase libraries and offers a wide variety of candidate glycosidases for application in food industry. Photosynthetic microorganisms (microalgae and cyanobacteria) serve as promising resources to excavate the intra- and extra-cellular β-galactosidases^13–15^. However, the reports about the biochemical characterization of new β-galactosidases from microalgae or cyanobacteria are still relatively few.

*Nostoc flagelliforme* is a soil surface-dwelling cyanobacterium inhabiting the xeric steppes of western China^16^. It exhibits a predominantly filamentous (hair-like or cylindrical) colony shape. In previous studies, an acidic water stress protein, WspA, was identified to be a novel β-galactosidase in *N. flagelliforme* and its close relative *Nostoc commune*^17,18^. WspA was synthesized in cells under ultraviolet irradiation or desiccation stress and secreted into extracellular polysaccharide matrix upon rehydration^19^. It was recently reported that *wspA* sequences showed high polymorphism in *N. flagelliforme* colonies^20^. In the sequenced *N. flagelliforme* CCNUN1 (NCBI BioProject, PRJNA407846), there are two adjacent *wspA* genes (*COO91_01770* and *COO91_01773*)^21^. In the sequenced *N. commune* HK-02and *Nostoc sphaeroides* CCNUC1, there is one (*NIES4070_53480*) and three (*GXM_06477*, *GXM_06476*, and *GXM_06474*) *wspA* genes, respectively. *wspA1* gene was first reported in *N. commune*^19^ and was also amplified by PCR in *N. flagelliforme* colonies^18^. Since the recombinant full-length WspA1 was always expressed as inclusion bodies in *E. coli* protein expression system, we had generated two truncated proteins of WspA1 (Wsp_A_ and Wsp_B_) for biochemical characterization^18^. The effects of temperature, pH, and metal ions on the activities of Wsp_A_ and Wsp_B_ as well as their catalytic constant *K*m were characterized in our previous study. The enzymatic activity of Wsp_A_ was stronger than that of Wsp_B._ However, there still remain some uncharacterized biochemical features for WspA1, such as the active form (monomer or multimer), enzymatic inhibitors, the active center, and so on. In addition, the potential homologs of the well-known β-galactosidase LacZ in *N. flagelliforme* have also not been characterized so far. In the present study, we identified a LacZ in *N. flagelliforme* (hereinafter Nf-LacZ) and conducted comparative biochemical analysis of Nf-LacZ and WspA1. Further, we focused on WspA1 to explore its central active region by using the protein truncation test. Besides, we investigated the possible role of the specific N-terminus of WspA1.

## Material and methods

### Cloning of β-galactosidase genes in *N. flagelliforme*

The potential β-galactosidase LacZ in *N. flagelliforme* was identified by local blasting (BioEdit software) against the proteome fasta file of *N. flagelliforme* CCNUN1 with the well-known *E. coli* (strain K12) LacZ (JW0335, KEGG)^9^. The resulting LacZ homolog is AUB41471 (NCBI), which is encoded by the gene *COO91_07519* (KEGG). *Nf-LacZ* sequence was amplified by PCR from genomic DNA of the *N. flagelliforme* culture in our laboratory. The NCBI accession no. for WspA1 is ABA54841 and its complete CDS can be retrieved from NCBI accession no. DQ155425. Various truncated sequences of *wspA1* were amplified by PCR from our previously constructed plasmid pMD18-T::*wspA1*^22^. The PCR primers used in this part (primer no. 1–9) were summarized in Table 1. PCR products were digested with the restriction endonucleases *Nde* I and *Bam*H I and constructed into the plasmid pET28a between the same restrictive sites. All the constructions were verified by sanger sequencing.

**Table 1:** The PCR primers used in this study.

Primer no., name	Primer sequence (5ˊ-3ˊ)	Primer combination
1, Nf-LacZ-F	ggaattcCATATGAAATTGTTGGATATACA	/
2, Nf-LacZ-R	cgGGATCCTTAACAGCTTGTGGGAGTACAT	1 + 2, for Nf-LacZ
3, Wsp_A_-F	ggaattcCATATGGTAGATCAGCCTTTTGCTCC	/
4, Wsp_A_-R	cgGGATCCTTATTCATTCACAATTGCAAAG	3 + 4, for Wsp_A_
5, Wsp_C1_-F	ggaattcCATATGACTACAGCTAATCCTGGAAC	/
6, Wsp_C1_-1R	cgGGATCCTTAACCTAAACCTAGACCTGATG	5 + 6, for Wsp_C1_
7, Wsp_C2_-2R	cgGGATCCTTAACCAGTTTCCAGCCAACCGA	5 + 7, for Wsp_C2_
8, Wsp_C3_-3R	cgGGATCCTTAATCTGCGTTGACATTCAAAG	5 + 8, for Wsp_C3_
9, Wsp_C4_-4R	cgGGATCCTTACTCACCAGTGAGCAGATCGA	5 + 9, for Wsp_C4_
10, WspA1-F	ATGTCGTTAAAGACTTTTAG	/
11, WspA1-R	TTCATTCACAATTGCAAAG	10 + 11, for WspA1::GFP
12, Wsp_B_-F	ATGGCTCTTTACGGCTATAC	12 + 11, for Wsp_B_::GFP
13, Wsp_N_-R	cccCTCGAGAATTTTCTCCTTATTGAA	10 + 13, for Wsp_N_::GFP

The protective bases in the primers were shown in lowercase letters.

### In vitro expression and purification

The *E. coli* BL21(DE3)/pET28a protein expression system (Novagen, USA) was used to express target proteins. The above constructs were transformed into the *E. coli* stain to produce target proteins with His-tags at the N-terminus. The transformed *E. coli* strains were grown in 200 ml LB medium (containing 50 μg/ml kanamycin) at 37 °C and 220 rpm until the optimum density at 600 nm (OD_600_) reached up to 0.5 ~ 0.6, and then the cultures were subjected to protein induction for 6 h with 0.2 mM Isopropyl β-D-thiogalactoside (IPTG). After centrifugation, the pellets were crushed by a low-temperature high-pressure crusher. The crude proteins were loaded on Ni His•Bind resin gravity column (Novagen, USA). The column was washed with the buffer (20 mM Tris–HCl, 500 mM NaCl, 80 mM imidazole, 5% glycerol, pH 8.0) to remove unwanted proteins and then the target protein was eluted with the buffer (20 mM Tris–HCl, 500 mM NaCl, 1000 mM imidazole, 5% glycerol, pH 8.0). Protein profiling or separation was examined using 12% sodium dodecyl sulfate–polyacrylamide gel electrophoresis (SDS-PAGE)^23^. If necessary, eluted proteins were further purified by gel filtration with fast protein liquid chromatography (FPLC) system (AKTA purifier, GE Healthcare, Sweden), which was equipped with an anion exchange column HiTrap Q FF (16 × 25 mm, GE Healthcare)^24^. Protein concentration was determined with the Bradford assay^25^.

### Protein polymerization assay

The gel filtration with the FPLC system can be also employed to analyze the polymerization state of native or active proteins^26^. Protein samples were injected into the column for separation, which was equilibrated with the buffer (20 mM Tris–HCl, 150 mM NaCl, 10% glycerol, pH 7.5). Five molecular weight markers were used: beta-amylase (200 kDa), alcohol dehydrogenase (150 kDa), albumin (66 kDa), carbonic anhydrase (29 kDa), and cytochrome c (12.4 kDa) (GE Healthcare, China agency). Protein separation was monitored by measuring the absorbance at 280 nm. To analyze the effects of acidic or more alkaline conditions on the protein polymerization state, the above-mentioned buffer was adjusted to pH 5.5 and 8.5, respectively.

### Galactosidase activity assay

Galactosyl hydrolytic and transgalactosylation activities of the target proteins were assayed as previously described^18^ with slight modifications. Galactosyl hydrolytic activity was assayed in 1 ml of 0.1 M phosphate-buffered saline (PBS) solution (pH 7.5) with 3 mM o-nitrophenyl-β-D-galactopyranoside (ONPG) as the substrate. The final protein concentration used for the reaction was 10 µg/ml. The reactions were conducted at 37 °C and stopped by supplementation with 100 μl of 1 M Na_2_CO_3_ solution. The absorbance of the reaction product o-nitrophenol (ONP) was measured at 405 nm.

Transgalactosylation reactions were performed at 37 °C for 3 h by incubation of 20 μg/ml enzyme (final concentration) with 20 μl of the acceptor glucose (500 mM) and 60 μl of ONPG (50 mM) in 100 μl of 0.1 M PBS buffer (pH 7.5). Products of the transgalactosylation reaction were examined by thin-layer chromatography (TLC)^27^. Each reaction solution of 2 μl was dropped on the silica gel plate for TLC analysis with methanol:chloroform (40:60) as the mobile phase. After the chromatography, the plate was air-dried at room temperature. The chromatogram observation was performed by spraying 20% H_2_SO_4_ on the silica gel plate and heating at 115 °C for 15 min.

### Enzymatic inhibitor assay

Enzymatic inhibition reaction was conducted in the above-mentioned galactosyl hydrolytic solution by supplementing various concentrations of glycosidase inhibitors. Four inhibitors were used: 4-methylumbelliferyl-beta-D-glucopyranoside (4-MU-Glu), conduritol B epoxide (CBE), acarbose, and galactostatin bisulfite (GBS) (ALFA Chemistry, USA). The reaction was conducted at 37 °C. The protein concentration was 10 µg/ml. Similarly, the absorbance at 405 nm of the reaction product was measured. For evaluating the inhibitory effects of these inhibitors, the concentration range of 0 ~ 200 µM and the extended reaction time of 9 h were considered in the initial test.

The half-maximal inhibitory concentration (IC50) represents the concentration of a substance (e.g. a drug) that is required for 50% inhibition in a specific biological or biochemical function^28^. The IC50 values of the galactosidases in response to GBS inhibition were assayed as previously described^29^ with slight modification. For IC50 detection, the reaction was conducted at 37 °C for 3 h, with the concentrations of GBS ranging from 0 to 50 µM.

### Expression of GFP-fused proteins in *Nostoc* sp. PCC 7120

Three nucleotide sequences, *wspA1*, the N-terminal sequence of *wspA1* (*wsp*_*N*_), and the truncated sequence of *wspA1* without *wsp*_*N*_ (*wsp*_*B*_), were amplified by PCR from the plasmid pMD18-T::*wspA1* with the primers (primer no. 10–13) as shown in Table 1. For generating green fluorescent protein (GFP) gene-fused constructions, a plasmid pRL25C-GFP^30^ was modified by introducing a *petE* promoter^31^ and two adjacent restriction sites *Sma* I and *Xho* I, and then the PCR products were inserted into the modified plasmid. Plasmid transformation and transformant selection were performed as previously described^32^. GFP fluorescence signals in the transgenic cells were observed by confocal laser-scanning microscopy (Leica, Germany). GFP was excited at 488 nm by an argon-ion laser.

### Western blotting analysis

The above transgenic cells at the exponential period (OD_750_ of 0.4 ~ 0.6) were collected by centrifugation at 6,000 rpm for 5 min. The pelleted cells were subjected to protein extraction and the crude protein extracts were separated on 12% SDS-PAGE gels for western blotting as previously described^22^. Anti-WspA1 rabbit antiserum was used for the blotting. In addition, the remaining supernatants were filtered with double filter papers to remove residual cells. The initial chlorophyll fluorescence (*F*_*0*_) of possibly residual cells in the filtered solutions was detected by a plant efficiency analyzer (Hansatech Instruments Ltd., UK)^33^. The *F*_*0*_ value of zero confirmed no cell contamination. The solutions were freeze-dried and the pellets (containing the released proteins) were subjected to western blotting as the above-mentioned.

### Phylogenetic analysis

The Nf-LacZ sequence was used to query the KEGG database with BLAST, and the resulting top 50 hits were retrieved from the database. The LacZ from *E. coli* MS 85–1 (b0344, KEGG) was used as an outgroup. The amino acid sequences were aligned using mafft^34^. The resulting sequence alignments were adjusted using trimAl^35^ by removing spurious sequences. The maximum-likelihood phylogenetic tree was inferred using IQ-Tree 2.1.2^36^ with the LG + F + R5 model and 1,000 bootstraps.

## Results

### Identification of the β-galactosidase LacZ in N. flagelliforme

**Figure 1:**
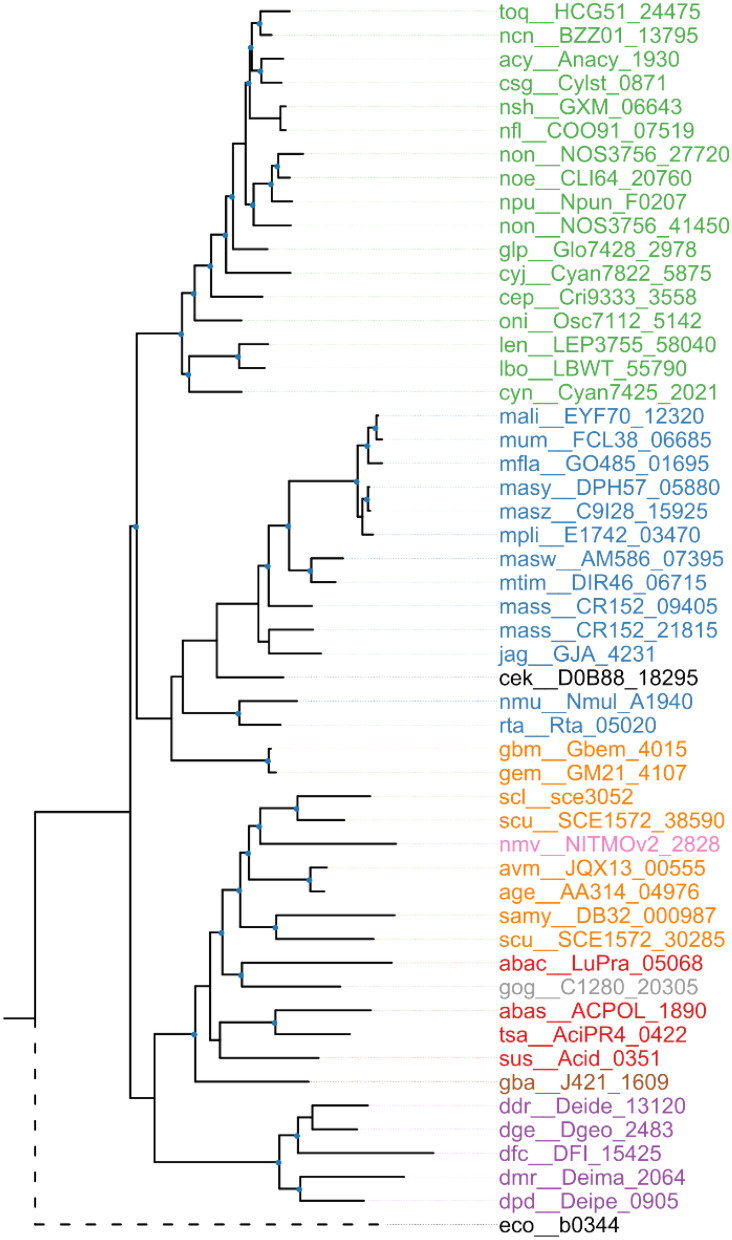
Phylogenetic analysis of Nf-LacZ (COO91_07519) with its top 50 similar homologs from the KEGG database. The nodes with bootstrap values higher than 70% were highlighted with blue dots. The lacZ homologs from Cyanobacteria (green) form a distinct clade. The homologs from Betaproteobacteria were highlighted in blue, the homologs from Deltaproteobacteria were highlighted in orange, the homologs from Acidobacteria were highlighted in red, and the homologs from Deinococcus-Thermus group were highlighted in purple. The species names for these proteins were included in supplemental table S1.

The potential homologs of the β-galactosidase LacZ have not yet been identified in *N. flagelliforme.* In this study, a putative Nf-LacZ (COO91_07519) was identified as described in the methods. Nf-LacZ consists of 619 amino acid residues with a calculated molecular weight of 70.8 kDa. The Pfam domain analysis showed that Nf-LacZ possesses the TIM barrel domain and the sugar-binding domain of GH family 2. Phylogenetic analysis suggested that LacZ homologs from cyanobacterial species form a distinct clade (Fig. 1).

**Figure 2:**
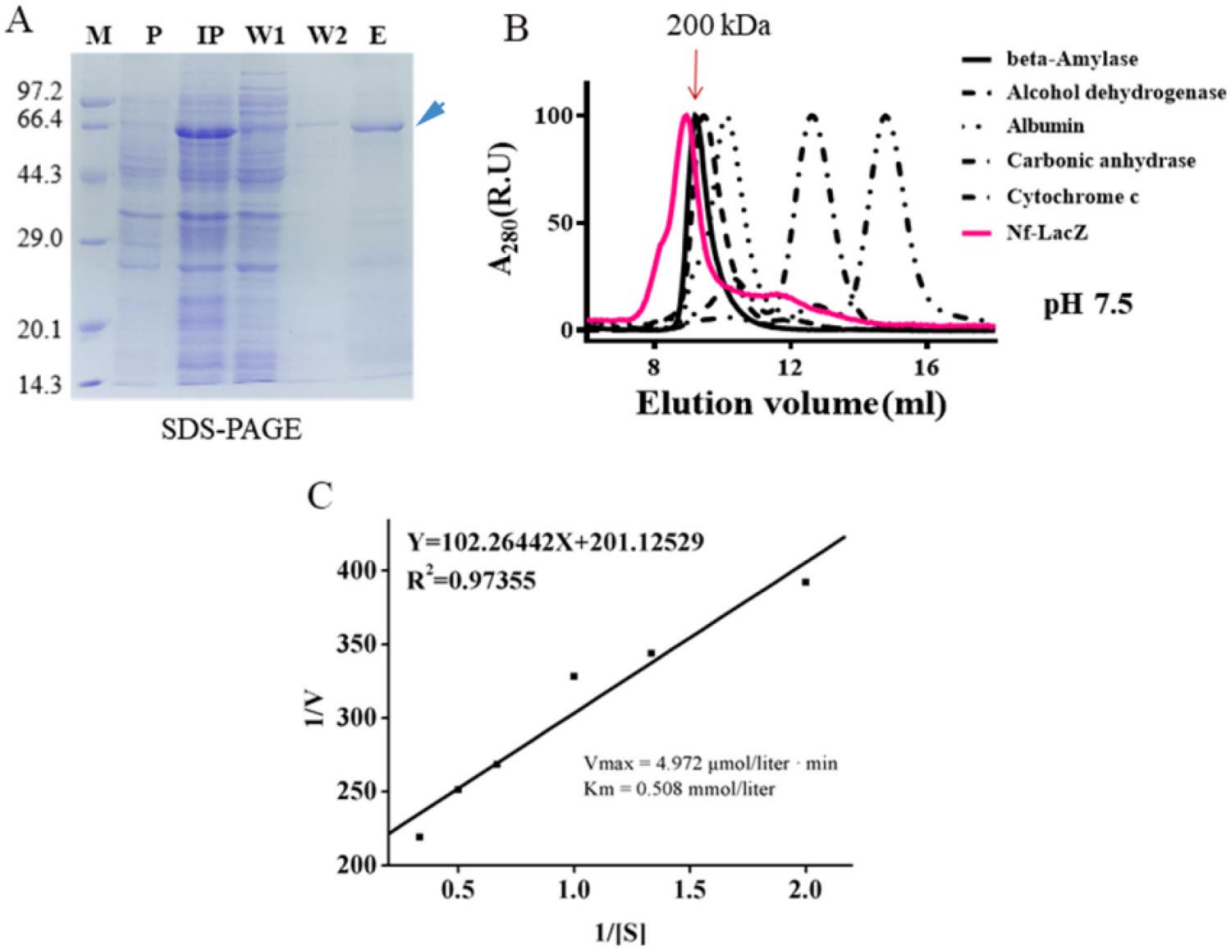
In vitro expression and enzymatic analysis of Nf-LacZ protein. (**A**) In vitro expression of Nf-LacZ by the *E. coli* BL21/pET28a protein expression system. *M* marker protein, *P* total proteins before IPTG induction, *IP* total proteins after IPTG induction, *W1 and W2* washed fractions, *E* eluted fraction. Blue arrow points to the Nf-LacZ protein. (**B**) The protein polymerization state analyzed by FPLC. Molecular weight markers: beta-amylase, 200 kDa; alcohol dehydrogenase, 150 kDa; albumin, 66 kDa; carbonic anhydrase, 29 kDa; cytochrome c, 12.4 kDa. (**C**) Michaelis kinetic analysis. *V*_*max*_ and *K*_*m*_ values were calculated. ONPG serves as the substrate. Reaction was conducted at 45 °C and pH 8.0.

The recombinant Nf-LacZ was expressed by employing the *E. coli* expression system. As shown in the SDS-PAGE gel, Nf-LacZ was effectively induced and then separated (Fig. 2A). Gel filtration with FPLC is often used to analyze or purify mixtures of proteins according to size and charge^37^. Based on the molecular weight markers in this FPLC analysis (Fig. 2B), native Nf-LacZ should be a multimeric protein (at least a trimer or larger). The effects of pH and temperature on the enzymatic activity were also assayed using ONPG as a substrate. The optimum temperature and pH for Nf-LacZ are 40 °C and pH 6.5, respectively (Supplemental Fig. S1). Metal ions may also affect the activity of the β-galactosidase. It was found that the metal ions, K^+^, Mg^2+^, Ca^2+^, Zn^2+^, and Mn^2+^ can all enhance the enzymatic activity of Nf-LacZ (Supplemental Fig. S1). In contrast, the optimum temperature and pH for Wsp_A_ are 45 °C and pH 8.0, respectively, and Ca^2+^ and Zn^2+^ are inhibitory for the activity of Wsp_A_^18^. Further, the kinetic parameters *K*_*m*_ and *V*_*max*_ of Nf-LacZ were determined with ONPG as the substrate (Fig. 2C). The *Km* value was 0.5 mmol/liter for Nf-LacZ, which is close to that of Wsp_A_^18^. Thus, Nf-LacZ has a similar affinity as Wsp_A_ for ONPG under the tested condition.

### Analysis of the polymerization of native Wsp_A_

**Figure 3:**
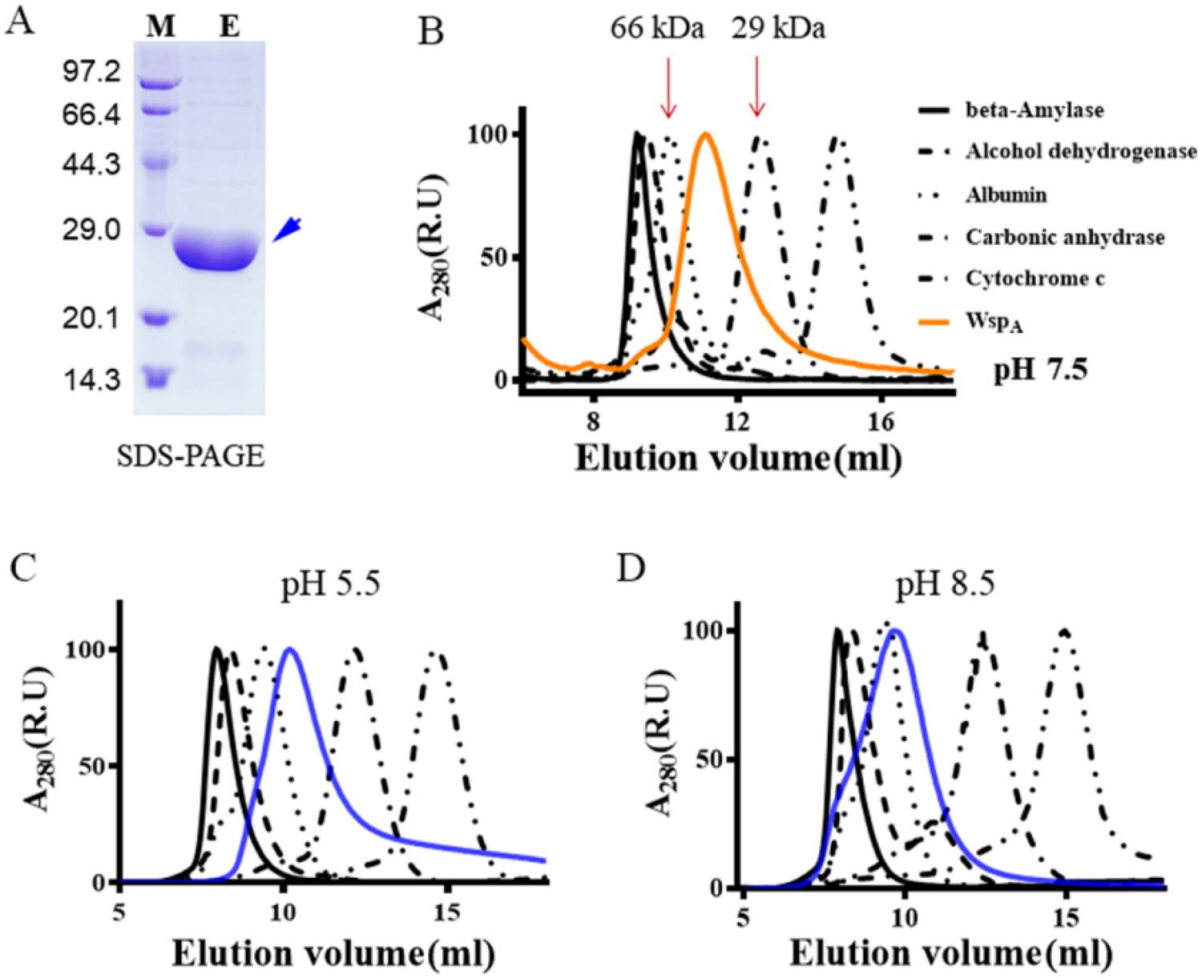
In vitro expression of Wsp_A_ and analysis of the protein polymerization state. (**A**) In vitro expression of Wsp_A_ by the *E. coli* BL21/pET28a protein expression system. *M* marker protein, *E* eluted fraction. Blue arrow points to the target protein. (**B**) The protein polymerization state of native Wsp_A_ analyzed by FPLC at pH 7.5. (**C**) The effect of unfavorable acidic condition (pH 5.5) on the dimer of Wsp_A_. (**D**) The effect of unfavorable alkaline condition (pH 8.5) on the dimer of Wsp_A_.

Wsp_A_ consists of 234 amino acid residues with a calculated molecular weight of 24.0 kD. The polymerization state of native Wsp_A_ was not explored in our previous study. As shown in the SDS-PAGE gel, the recombinant Wsp_A_ that was expressed by the *E. coli* expression system was separated (Fig. 3A). Subsequently, the polymerization state of native Wsp_A_ was analyzed by FPLC (Fig. 3B). FPLC fraction of Wsp_A_ was between the 29 and 66 kDa makers, implying that native Wsp_A_ is not a monomer but a dimer. Our previous study showed that Wsp_A_ had a narrow optimal pH range; at pH 5.5, the activity of Wsp_A_ reduced to nearly zero, while at pH 8.5 the activity decreased more than 60% compared to the maximum activity at pH 8.0^18^. However, FPLC analysis showed that the dimer of Wsp_A_ was not dissociated at both pH 5.5 (Fig. 3C) and pH 8.5 (Fig. 3D). Therefore, native Wsp_A_ forms a stable dimer although its activity can be affected by the unfavorable acid–base environment.

### Effects of the inhibitors on the activities of Nf-LacZ and Wsp_A_

**Figure 4:**
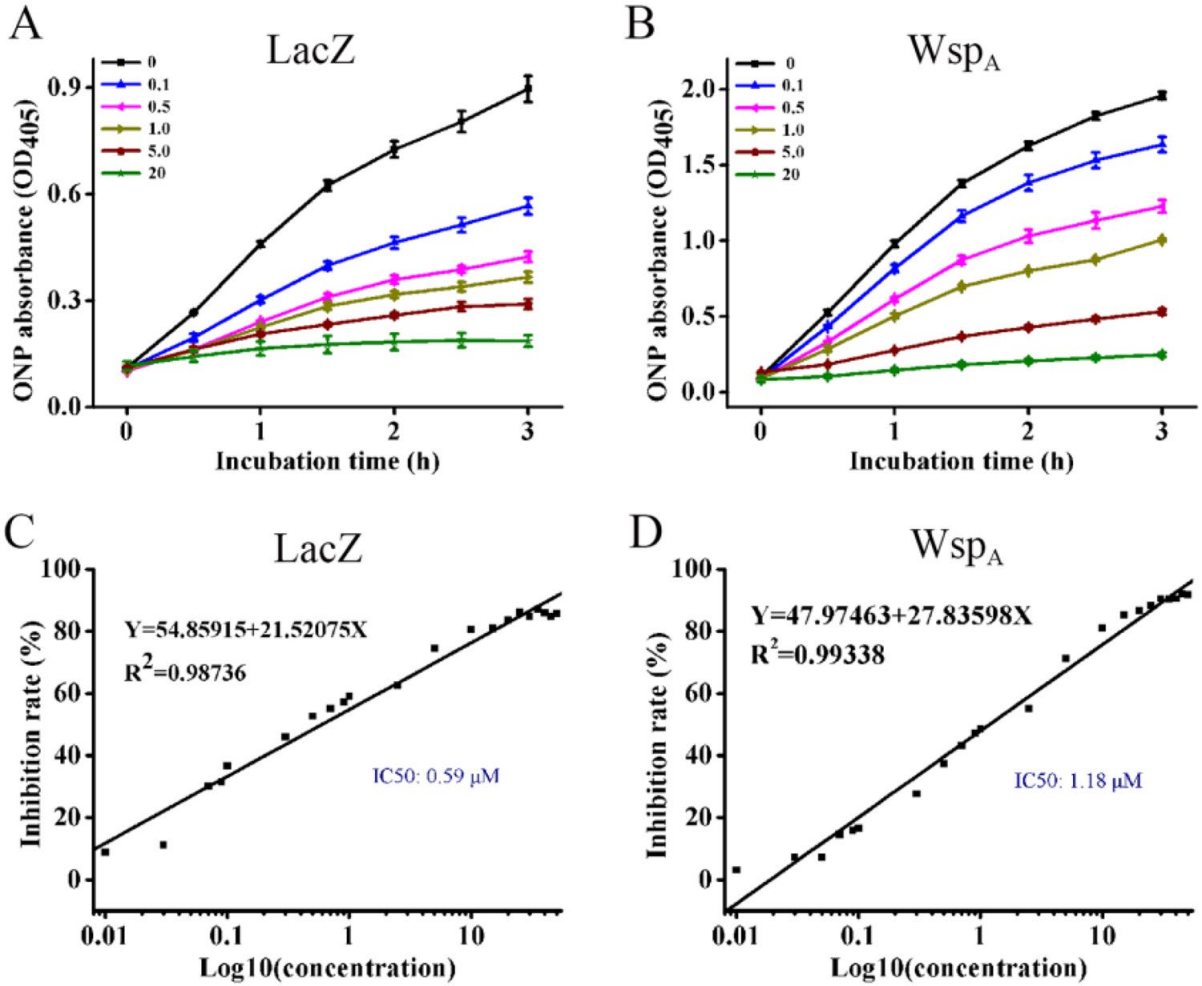
The inhibitory effects of GBS on the activities of Nf-LacZ and Wsp_A_. (**A**,**B**), the galactosyl hydrolytic reactions of Nf-LacZ and Wsp_A_ in presence of different concentrations of GBS, respectively. GBS concentration: 0 ~ 20 µM. The absorbance of the reaction product ONP at 405 nm (OD_405_) was detected. Data shown are means ± SD (*n* = 3). (**C**,**D**), determination of the IC50 values of GBS inhibition on the activities of Nf-LacZ and Wsp_A_, respectively. GBS concentration ranged from 0.01 µM to 50 µM in the tests.

The response of the glycosidase to various inhibitors is an important aspect for characterization. The influences of glycosidase inhibitors on the activities of Nf-LacZ and Wsp_A_ were investigated. Totally four inhibitors, 4-MU-Glu, CBE, acarbose, and GBS, were used for testing. Among them, 4-MU-Glu, CBE, and acarbose did not show obvious inhibitory effects on both enzymes. The inhibitory effects of GBS on the two enzymes were then compared (Fig. 4). The activities of both Nf-LacZ (Fig. 4A) and Wsp_A_ (Fig. 4B) were markedly inhibited by 0.1 µM GBS. The IC50 value is widely used as the informative measure of an enzyme inhibitor’s efficacy^28^. The results showed that Nf-LacZ had an IC50 value of 0.59 µM (Fig. 4C), while Wsp_A_ had a IC50 value of 1.18 µM (Fig. 4D). Thus, Wsp_A_ was relatively less sensitive to the inhibitor GBS at the tested condition.

### Identification of the central activity region of WspA1

**Figure 5:**
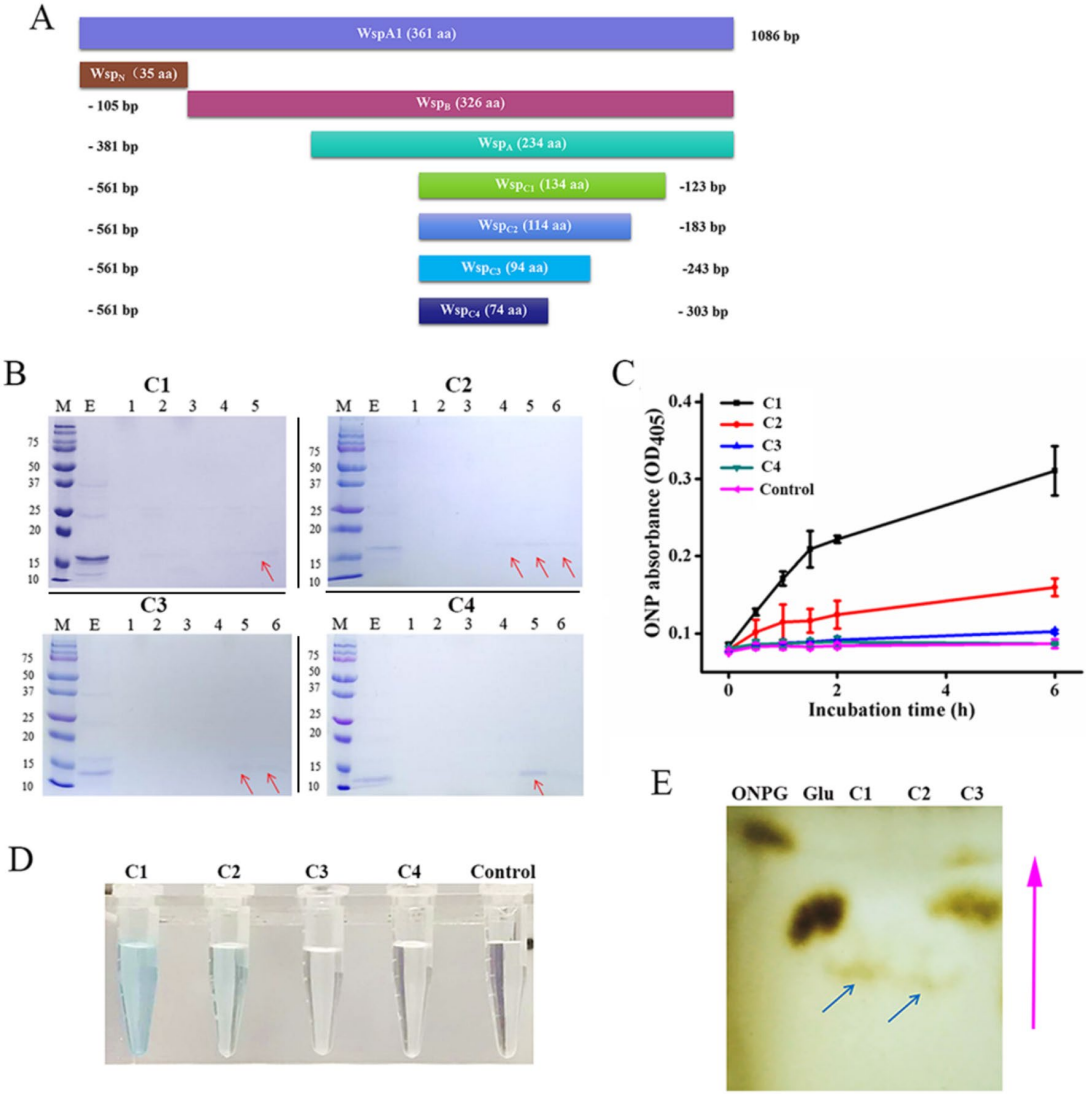
Biochemical analysis of the truncated proteins of WspA1. (**A**) An illustration of the truncated protein variants. (**B**) Protein profiles of the in vitro expressed and purified target proteins in SDS-PAGE gels. *M* marker protein, *E* eluted fraction. No. 1–6, the purified fractions by FPLC. The red arrows indicate the target proteins. C1, C2, C3 and C4 represent Wsp_C1_, Wsp_C2_, Wsp_C3_, and Wsp_C4_, respectively. (**C**) Comparative analysis of hydrolytic activities in 0.1 M PBS buffer (pH 7.5) with ONPG as the substrate. Data shown are means ± SD (*n* = 3). Protein concentrations, 10 μg/ml. ONPG, 3 mM. Control, no addition of any protein in the reaction buffer. (**D**) In vitro activity analysis of the four proteins in X-Gal buffer (pH 7.5). Similar conditions were used as in (**C**). Reactions were performed for 6 h. (**E**) TLC analysis of the transgalactosylation activity of the truncated proteins. Glucose was used as the acceptor. Blue arrows indicate the generated products. The pink arrow indicates the solvent diffusion direction.

The recombinant WspA1 was always expressed as inclusion bodies in *E. coli* cell and thus expression of its truncated proteins was one way to explore its biochemical functions^18^. To investigate the central activity region of WspA1, we designed four truncated WspA1 variants (Wsp_C1_, Wsp_C2_, Wsp_C3_, and Wsp_C4_) in this study (Fig. 5A), roughly according to its secondary structure predicted by PSIPRED^38^. The four truncated proteins were in vitro expressed and purified (Fig. 5B). Their catalytic features as a β-galactosidase were assayed using ONPG and 5-bromo-4-chloro-3-indolyl-β-D-galactoside (X-Gal) as the substrates. The biochemical analysis showed that Wsp_C1_, Wsp_C2_, and Wsp_C3_ had a successively decreased galactosyl hydrolytic activity, while Wsp_C4_ did not show the activity (Fig. 5C,D). Further, the potential transgalactosylation activities of Wsp_C1_, Wsp_C2_, and Wsp_C3_ were assayed by TLC with glucose as the acceptor (Fig. 5E). The result showed that the disaccharide or oligosaccharide was produced under the catalysis of Wsp_C1_ and Wsp_C2_, while Wsp_C3_ had no this catalytic activity. Therefore, Wsp_C2_ should represent the minimum active region of WspA1 with both hydrolytic and transgalactosylation activities up to now.

### Analysis of the role of the N-terminus of WspA1 in secretion

**Figure 6:**
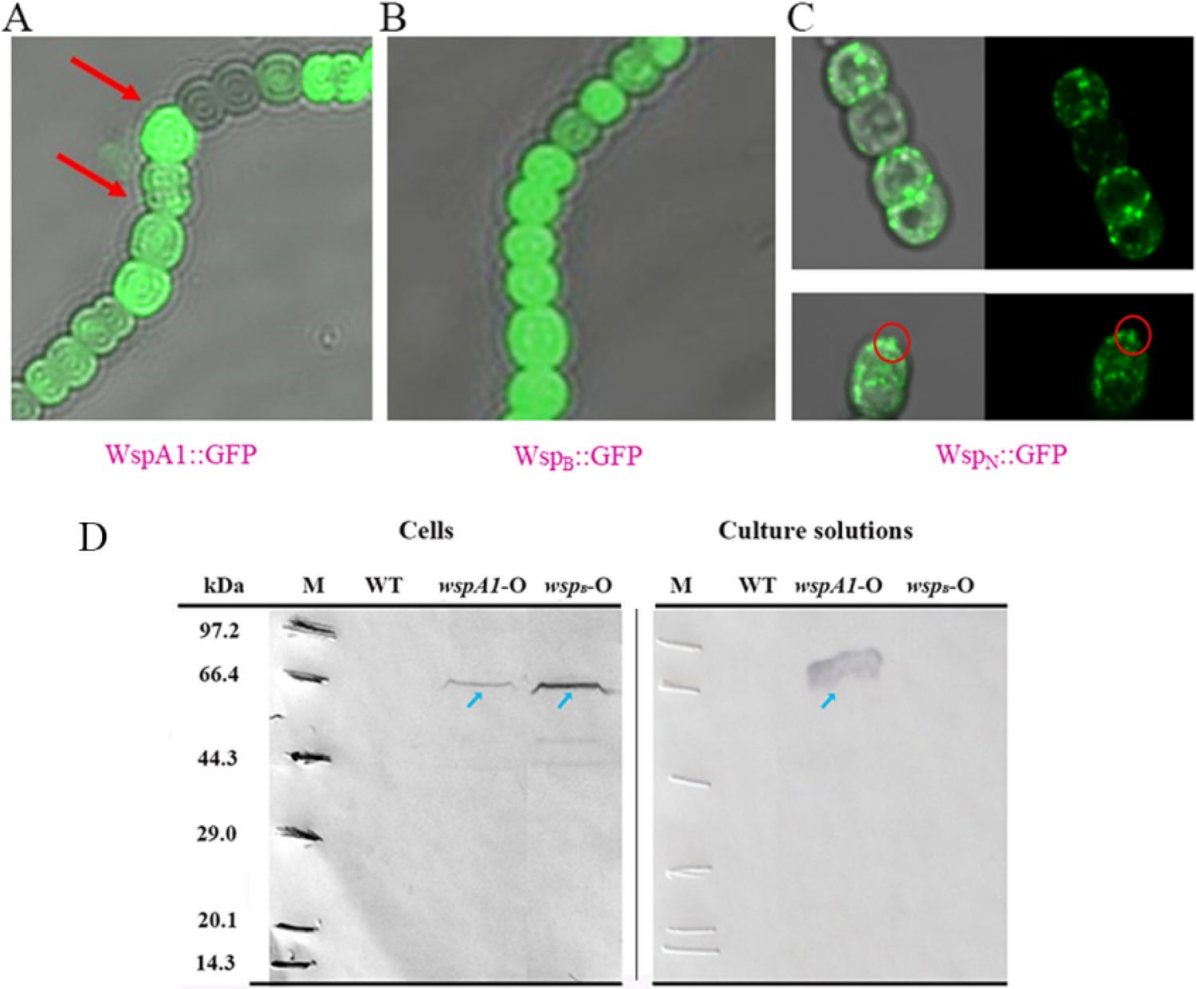
Examination of the potential export role of Wsp_N_ in transgenic *Nostoc* sp. PCC 7120 cells. Confocal microscopy observation of the GFP-fused proteins, WspA1::GFP (**A**), Wsp_B_::GFP (**B**), and Wsp_N_::GFP (**C**) in transgenic cells. For panel (**A**,**B**), a 40 × objective lens was used; for panel (**C**), a 60 × objective lens was used. Red arrows point to the secreted fluorescent foci; Red circles indicate the fluorescent foci that are seemingly in the process of secretion. (**D**) Western blotting analysis of WspA1::GFP and Wsp_B_::GFP proteins in transgenic cells and culture solutions. An anti-WspA1 antibody was used. Blue arrows point to the target proteins. WT, wild-type cells.

As implied in our previous attempts, the N-terminus of WspA1 (Wsp_N_) was a potential cause for the forming of inclusion bodies in the *E. coli* expression system. We further speculated that Wsp_N_ might have a potential role in facilitating the export of WspA1 from cells, since WspA can be secreted into extracellular polysaccharide matrix upon rehydration^19^. WspA1, Wsp_B_ (the WspA1 protein lacking Wsp_N_), and Wsp_N_ were respectively fused with the GFP protein, and their features regarding extracellular transport were examined in transgenic cells by confocal microscopy (Fig. 6A–C). In WspA1::GFP cells, sporadic fluorescent foci were observed in the periplasmic space (Fig. 6A), while no such fluorescent foci were observed in Wsp_B_::GFP cells (Fig. 6B). In Wsp_N_::GFP cells, fluorescent foci were scattered in cells and some of them seemed to be in the process of secretion (Fig. 6C). In addition, the crude proteins that were extracted from WspA1::GFP and Wsp_B_::GFP cells and their culture solutions were subjected to western blotting using anti-WspA1 antibody (Fig. 6D). WspA1 was detected in both its transgenic cells and the culture solution, while Wsp_B_ was only detected in its transgenic cells. Together, these results implied that Wsp_N_ had a potential role in facilitating the secretion of WspA1.

## Discussion

Microbial β-galactosidases hold particular importance due to their wide applications in food industries. They are also important tools for glycosylation of vital molecules in the medicine and cosmetic industries^7^. Characterization of new β-galactosidases from natural resources can enrich glycosidase libraries. This study conducted comparative characterization of two β-galactosidases Nf-LacZ and WspA1 from the terrestrial cyanobacterium *N. flagelliforme*, with more focus on the latter based on the previous research^18^. The LacZ homologs from some cyanobacteria form a distinct clade (Fig. 1). Biochemical analysis verified that Nf-LacZ functions as a β-galactosidase. However, Nf-LacZ shares only 25.2% sequence identity (Query coverage, 75%; *E*-value, 4e−21) with *E. coli* LacZ (JW0335). The *Km* values for *E. coli* LacZ with ONPG as the substrate ranged from 0.12 to 0.82 mmol/liter at specific conditions^39–41^. The *Km* value (0.5 mmol/liter) of Nf-LacZ falls in that range. The active form of *E. coli* LacZ is a tetramer^9^. According to the gel filtration assay, the size of native Nf-LacZ was larger than 200 kDa, implying that it is at least a trimer or larger. Its precise active form remains to be clarified.

By employing *E. coli* cell as a host, expression and production of recombinant proteins are not always successful and sometimes lead to form inclusion bodies^42^. The case is same for the full-length WspA1 protein and thus the protein truncation strategy was considered in the in vitro expression. The truncated proteins of WspA1 without the N-terminus (Fig. 5A) could be all obtained in soluble state. In most cases, we used Wsp_A_ for biochemical characterization. As indicated by the gel filtration assay, native WspA1 should be a dimer and pH alteration cannot dissociate the dimer. Cold-active β-galactosidases are an attractive group identified in low temperature-adapted microorganisms^10^. Two cold-active β-galactosidases from *Paracoccus* sp. 32d and *Arthrobacter* sp. 32cB are also dimers in their native form^43,44^. WspA1 has no significant sequence similarity to the two enzymes. WspA1 and its homologs are found in some colonial *Nostoc* species, including *N. flagelliforme*, *N. commune*, *Nostoc sphaeroides* and *Nostoc verrucosum*^18,19,45^. Thus, WspA proteins may also represent a novel group of β-galactosidase.

Nf-LacZ and Wsp_A_ have other different biochemical features. The optimum temperatures for the two enzymes are 40 °C and 45 °C, respectively, and both are very sensitive to higher temperature. The optimum pH values for them are 6.5 and 8.0, respectively, but Nf-LacZ seems more resistant to lower pH than Wsp_A_^18^ (Supplemental Fig. S1). The pH value of extracellular polysaccharide matrix is around 7.6 in *N. flagelliforme*^46^, which may guarantee that the secreted WspA could function effectively in the matrix. In contrast, Nf-LacZ is an intracellular protein in *N. flagelliforme*, since we did not detect this protein by mass spectrometry analysis of the exoproteins. The activities of both enzymes are stimulated by Mg^2+^, but Ca^2+^ is inhibitory for Wsp_A_. An in vitro experiment found that WspA could bind the UV-A/B absorbing pigment scytonemin through non-covalent interactions^19^. It implied that the activity of WspA might also be affected by the scytonemin molecule in the extracellular polysaccharide matrix. In addition, it was found that Nf-LacZ and Wsp_A_ have similar *Km* values, but the latter is less sensitive to the inhibitor GBS.

Our previous study showed that the activity of Wsp_B_ (Fig. 5A) was lower than that of Wsp_A_^18^. A derived question is which sequence region or domain in WspA1 is critical for the activity. The truncation test of WspA1 indicated that Wsp_C2_ (114 aa) can be recognized as the minimum central region with glycosyl hydrolytic and transgalactosylation activities. The smaller Wsp_C3_ (94 aa) has a very weak glycosyl hydrolytic activity, which implies that it might be the primitive sequence for the evolution of WspA1. Searching Wsp_C3_ against the NCBI nr database showed that this sequence was highly conserved (Supplemental Fig. S2). The species/strains having WspA homologs share a common feature of dense extracellular polysaccharide matrix. WspA was suggested to play a crucial role in the regulation of structural dynamics of the polysaccharide matrix for coping with periodic desiccation^18^. Thus, the present protein truncation analysis of WspA1 would advance our understanding on the evolution and function of WspA in those glycan-rich *Nostoc* species.

As the above mentioned, Wsp_N_ was prone to cause the forming of inclusion bodies in the *E. coli* expression system. We had speculated that Wsp_N_ might have a potential role in facilitating the export of WspA1 from cells. Our results showed that WspA1::GFP and Wsp_N_::GFP could be secreted from the cell in the form of small particles (fluorescent foci), while Wsp_B_::GFP could not (Fig. 6). The forming of secreted particles was also observed in our previous study in which WspA1::GFP transgenic *Arabidopsis* plants were generated^22^. Wsp_N_ is not a typical signal peptide as predicted by SignalP^47^. Thus, Wsp_N_ may represent a special or atypical transport way. The membrane-fusion potential of Wsp_N_ might also be an important reason for the forming of insoluble WspA1 in the *E. coli* expression system. Longer or shorter similar sequences of Wsp_N_ can be found in several other WspA homologs, AHB33430, QFS48983, QFS48982, and WP_100897955 (NCBI; Supplemental Fig. S2). However, it was also reported that the two WspA proteins (AUB35877 and AUB35880, NCBI) could be released from the cells of a *N. flagelliforme* culture but both proteins lack the Wsp_N_ sequence^48^. Thus, Wsp_N_-facilitated export may be an evolving new way for protein secretion.

**Figure 7:**
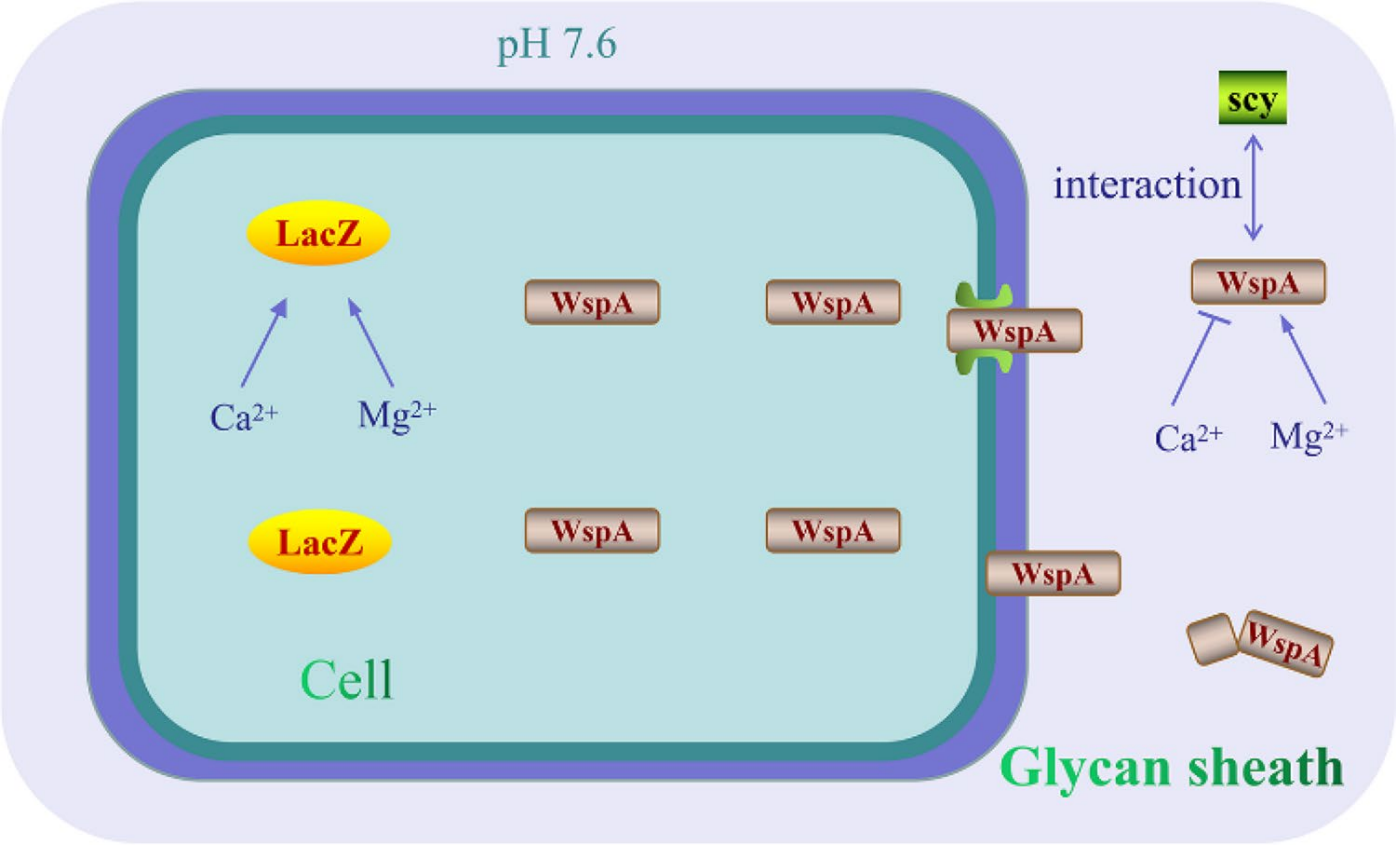
An illustration of the two β-galactosidases LacZ and WspA in a cell of *N. flagelliforme*. LacZ is located intracellularly. WspA is stored intracellularly, but can be secreted into the glycan sheath upon rehydration. The activities of both enzymes are promoted by Mg^2+^, while Ca^2+^ is inhibitory for WspA. In the glycan sheath, the activity of WspA may also be affected by the extracellular pigment scytonemin and its own hydrolysis. *scy* scytonemin.

The secreted WspA accounts for only a very minor part of the total WspA protein in the cells of *N. flagelliforme* and *N. commune*^18,19^. Also, it can be released from the desiccated colonies upon rehydration. In contrast, Nf-LacZ should be still a traditional intracellular β-galactosidase but with low sequence similarity with the well-known *E. coli* LacZ. An illustration of the two β-galactosidases, LacZ and WspA, in the *N. flagelliforme* cell is shown in Fig. 7.

In conclusion, we characterized some biochemical features of the two β-galactosidases Nf-LacZ and WspA1 from *N. flagelliforme*. They have different enzymatic characteristics and can serve as potential biocatalysts for use in food industry. Elucidation of the central active region of WspA1 provides a valuable clue for understanding its evolution. The future resolution of their crystal structures will provide more functional information.

## Supplementary Information

Supplementary Information.

## References

[1] Saqib S, Akram A, Halim SA, Tassaduq R (2017). Sources of β-galactosidase and its applications in food industry. 3 Biotech..

[2] Movahedpour A (2021). β-Galactosidase: From its source and applications to its recombinant form. Biotechnol. Appl. Biochem..

[3] Husain Q (2010). Beta galactosidases and their potential applications: A review. Crit. Rev. Biotechnol..

[4] Benešová E, Šućur Z, Těšínský M, Spiwok V, Lipovová P (2021). Transglycosylation abilities of β-d-galactosidases from GH family 2. 3 Biotech..

[5] Cheng W (2018). Effects of a galacto-oligosaccharide-rich diet on fecal microbiota and metabolite profiles in mice. Food Funct..

[6] Chandrasekar B, van der Hoorn RA (2016). Beta galactosidases in *Arabidopsis* and tomato—a mini review. Biochem. Soc. Trans..

[7] Lu L, Guo L, Wang K, Liu Y, Xiao M (2020). β-Galactosidases: A great tool for synthesizing galactose-containing carbohydrates. Biotechnol. Adv..

[8] Bartesaghi A, Matthies D, Banerjee S, Merk A, Subramaniam S (2014). Structure of β-galactosidase at 3.2-Å resolution obtained by cryo-electron microscopy. Proc. Natl. Acad. Sci. USA.

[9] Juers DH, Matthews BW, Huber RE (2012). LacZ β-galactosidase: Structure and function of an enzyme of historical and molecular biological importance. Protein Sci..

[10] Mangiagalli M, Lotti M (2021). Cold-active β-galactosidases: Insight into cold adaption mechanisms and biotechnological exploitation. Mar. Drugs.

[11] Higuchi Y (2018). Identification and characterization of a novel β-D-galactosidase that releases pyruvylated galactose. Sci. Rep..

[12] Carneiro LABC, Yu L, Dupree P, Ward RJ (2018). Characterization of a β-galactosidase from *Bacillus subtilis* with transgalactosylation activity. Int. J. Biol. Macromol..

[13] Zanette CM, Mariano AB, Yukawa YS, Mendes I, Spier MR (2019). Microalgae mixotrophic cultivation for β-galactosidase production. J. Appl. Phycol..

[14] Bentahar J, Doyen A, Beaulieu L, Deschênes JS (2019). Investigation of β-galactosidase production by microalga *Tetradesmus obliquus* in determined growth conditions. J. Appl. Phycol..

[15] Brasil BSAF, Siqueira FG, Salum TFC, Zanette ZM, Spier MR (2017). Microalgae and cyanobacteria as enzyme biofactories. Alg. Res..

[16] Gao K (1998). Chinese studies on the edible blue-green alga, *Nostoc flagelliforme*: A review. J. App. Phycol..

[17] Morsy FM, Kuzuha S, Takani Y, Sakamoto T (2008). Novel thermostable glycosidases in the extracellular matrix of the terrestrial cyanobacterium *Nostoc commune*. J. Gen. Appl. Microbiol..

[18] Liu W, Cui L, Xu H, Zhu Z, Gao X (2017). Flexibility-rigidity coordination of the dense exopolysaccharide matrix in terrestrial cyanobacteria acclimated to periodic desiccation. Appl. Environ. Microbiol..

[19] Wright DJ (2005). UV irradiation and desiccation modulate the three-dimensional extracellular matrix of *Nostoc commune* (Cyanobacteria). J. Biol. Chem..

[20] Gao X, Xu H, Yuan X (2021). The overlooked genetic diversity in the dryland soil surface-dwelling cyanobacterium *Nostoc flagelliforme* as revealed by the marker gene *wspA*. Microb. Ecol..

[21] Shang JL (2019). Genomic and transcriptomic insights into the survival of the subaerial cyanobacterium *Nostoc flagelliforme* in arid and exposed habitats. Environ. Microbiol..

[22] Ai Y, Yang Y, Qiu B, Gao X (2014). Unique WSPA protein from terrestrial macroscopic cyanobacteria can confer resistance to osmotic stress in transgenic plants. World J. Microbiol. Biotechnol..

[23] Jones GL, Wilson ID (2000). ELECTROPHORESIS | One-Dimensional Sodium Dodecyl Sulfate Polyacrylamide Gel Electrophoresis. Encyclopedia of Separation Science.

[24] Yang YW (2019). Orange and red carotenoid proteins are involved in the adaptation of the terrestrial cyanobacterium *Nostoc flagelliforme* to desiccation. Photosynth. Res..

[25] Bradford MM (1976). A rapid and sensitive method for the quantitation of microgram quantities of protein utilizing the principle of protein-dye binding. Anal. Biochem..

[26] Irvine GB (2001). Determination of molecular size by size-exclusion chromatography (gel filtration). Curr. Protoc. Cell Biol..

[27] Raadsveld CW, Klomp H (1971). Thin-layer chromatographic analysis of sugar mixtures. J. Chromatogr. A.

[28] Georgakis N (2020). Determination of half-maximal inhibitory concentration of an enzyme inhibitor. Methods Mol. Biol..

[29] Dai G, Deblois CP, Liu S, Juneau P, Qiu B (2008). Differential sensitivity of five cyanobacterial strains to ammonium toxicity and its inhibitory mechanism on the photosynthesis of rice-field cyanobacterium Ge-Xian-Mi (*Nostoc*). Aquat. Toxicol..

[30] Zhang LC, Chen YF, Chen WL, Zhang CC (2008). Existence of periplasmic barriers preventing green fluorescent protein diffusion from cell to cell in the cyanobacterium *Anabaena* sp. strain PCC 7120. Mol. Microbiol..

[31] Gao X, Xu H, Zhu Z, She Y, Ye S (2020). Improved production of echinenone and canthaxanthin in transgenic *Nostoc* sp. PCC 7120 overexpressing a heterologous *crtO* gene from *Nostoc flagelliforme*. Microbiol. Res..

[32] Wolk CP, Vonshak A, Kehoe P, Elhai J (1984). Construction of shuttle vectors capable of conjugative transfer from *Escherichia coli* to nitrogen-fixing filamentous cyanobacteria. Proc. Natl. Acad. Sci. U S A.

[33] Zhao XM, Bi YH, Chen L, Hu S, Hu ZY (2008). Responses of photosynthetic activity in the drought-tolerant cyanobacterium, *Nostoc flagelliforme* to rehydration at different temperature. J. Arid Environ..

[34] Katoh K, Misawa K, Kuma K, Miyata T (2002). MAFFT: A novel method for rapid multiple sequence alignment based on fast Fourier transform. Nucleic Acids Res..

[35] Capella-Gutiérrez S, Silla-Martínez JM, Gabaldón T (2009). trimAl: A tool for automated alignment trimming in large-scale phylogenetic analyses. Bioinformatics.

[36] Minh BQ (2020). IQ-TREE 2: New models and efficient methods for phylogenetic inference in the genomic era. Mol. Biol. Evol..

[37] Madadlou A, O'Sullivan S, Sheehan D (2011). Fast protein liquid chromatography. Methods Mol. Biol..

[38] McGuffin LJ, Bryson K, Jones DT (2000). The PSIPRED protein structure prediction server. Bioinformatics.

[39] Huber RE, Parfett C, Woulfe-Flanagan H, Thompson DJ (1979). Interaction of divalent cations with beta-galactosidase (*Escherichia coli*). Biochemistry.

[40] Roth NJ, Huber RE (1996). The beta-galactosidase (*Escherichia coli*) reaction is partly facilitated by interactions of His-540 with the C6 hydroxyl of galactose. J. Biol. Chem..

[41] Xu J, McRae MA, Harron S, Rob B, Huber RE (2004). A study of the relationships of interactions between Asp-201, Na^+^ or K^+^, and galactosyl C6 hydroxyl and their effects on binding and reactivity of beta-galactosidase. Biochem. Cell Biol..

[42] Gräslund S (2008). Protein production and purification. Nat. Methods.

[43] Pawlak-Szukalska A, Wanarska M, Popinigis AT, Kur J (2014). A novel cold-active β-d-galactosidase with transglycosylation activity from the Antarctic *Arthrobacter* sp. 32cB–Gene cloning, purification and characterization. Proc. Biochem..

[44] Wierzbicka-Woś A (2011). A novel cold-active β-D-galactosidase from the *Paracoccus* sp. 32d–Gene cloning, purification and characterization. Microb. Cell Fact..

[45] Arima H (2012). Molecular genetic and chemotaxonomic characterization of the terrestrial cyanobacterium *Nostoc commune* and its neighboring species. FEMS Microbiol. Ecol..

[46] Gao X, Liu LT, Liu B (2019). Dryland cyanobacterial exopolysaccharides show protection against acid deposition damage. Environ. Sci. Pollut. Res..

[47] Almagro Armenteros JJ (2019). SignalP 5.0 improves signal peptide predictions using deep neural networks. Nat. Biotechnol..

[48] Yuan XL (2021). Investigations of solid culture-induced acquisition of desiccation tolerance in liquid suspension culture of *Nostoc flagelliforme*. J. Appl. Phycol..

