# Supplementary material for: Characterization of two β-galactosidases LacZ and WspA1 from Nostoc flagelliforme with focus on the latter’s central active region
Source: Sci Rep. 2021 Sep 16;11:18448. doi: 10.1038/s41598-021-97929-6 (PMC8445988; doi:10.1038/s41598-021-97929-6)
Supplement: Supplementary file 1 — Supplementary Information. [file 41598_2021_97929_MOESM1_ESM.pdf]

Supplemental materials for the paper entitled "Characterization of two β-galactosidases LacZ and WspA1 from *Nostoc flagelliforme* with focus on the latter's central active region"

Table S1 LacZ homologs used for phylogenetic analysis

ID	Gene ID	Group	Species Name
nfl__COO91_07519	COO91_07519	Cyanobacteria	*Nostoc flagelliforme*
nsh__GXM_06643	GXM_06643	Cyanobacteria	*Nostoc sphaeroides*
csg__Cylst_0871	Cylst_0871	Cyanobacteria	*Cylindrospermum stagnale*
ncn__BZZ01_13795	BZZ01_13795	Cyanobacteria	*Nostocales cyanobacterium* HT-58-2
acy__Anacy_1930	Anacy_1930	Cyanobacteria	*Anabaena cylindrica*
noe__CLI64_20760	CLI64_20760	Cyanobacteria	*Nostoc sp.* CENA543
toq__HCG51_24475	HCG51_24475	Cyanobacteria	*Tolypothrix* sp. PCC 7910
glp__Glo7428_2978	Glo7428_2978	Cyanobacteria	*Gloeocapsa* sp. PCC 7428
non__NOS3756_27720	NOS3756_27720	Cyanobacteria	*Nostoc sp.* NIES-3756
npu__Npun_F0207	Npun_F0207	Cyanobacteria	*Nostoc punctiforme*
non__NOS3756_41450	NOS3756_41450	Cyanobacteria	*Nostoc sp.* NIES-3756
cep__Cri9333_3558	Cri9333_3558	Cyanobacteria	*Crinalium epipsammum*
oni__Osc7112_5142	Osc7112_5142	Cyanobacteria	*Oscillatoria nigro-viridis*
cyj__Cyan7822_5875	Cyan7822_5875	Cyanobacteria	*Gloeothece verrucosa*
cyn__Cyan7425_2021	Cyan7425_2021	Cyanobacteria	*Cyanothece* sp. PCC 7425
lbo__LBWT_55790	LBWT_55790	Cyanobacteria	*Leptolyngbya boryana*
len__LEP3755_58040	LEP3755_58040	Cyanobacteria	*Leptolyngbya* sp. NIES-3755
cek__D0B88_18295	D0B88_18295	Gammaproteobacteria	*Cellvibrio* sp. KY-YJ-3
mtim__DIR46_06715	DIR46_06715	Betaproteobacteria	*Massilia oculi*
mass__CR152_09405	CR152_09405	Betaproteobacteria	*Massilia violaceinigra*
jag__GJA_4231	GJA_4231	Betaproteobacteria	*Janthinobacterium agaricidamnosum*
masw__AM586_07395	AM586_07395	Betaproteobacteria	*Massilia* sp. WG5
mass__CR152_21815	CR152_21815	Betaproteobacteria	*Massilia violaceinigra*
rta__Rta_05020	Rta_05020	Betaproteobacteria	*Ramlibacter tataouinensis*
gem__GM21_4107	GM21_4107	Deltaproteobacteria	*Geobacter* sp. M21
age__AA314_04976	AA314_04976	Deltaproteobacteria	*Archangium gephyra*
nmu__Nmul_A1940	Nmul_A1940	Betaproteobacteria	*Nitrosospira multiformis*
gbm__Gbem_4015	Gbem_4015	Deltaproteobacteria	*Geobacter bemidjiensis*
masz__C9I28_15925	C9I28_15925	Betaproteobacteria	*Massilia armeniaca*
masy__DPH57_05880	DPH57_05880	Betaproteobacteria	*Massilia* sp. YMA4
mfla__GO485_01695	GO485_01695	Betaproteobacteria	*Massilia flava*
mum__FCL38_06685	FCL38_06685	Betaproteobacteria	*Massilia umbonata*
mali__EYF70_12320	EYF70_12320	Betaproteobacteria	*Massilia albidiflava*
mpli__E1742_03470	E1742_03470	Betaproteobacteria	*Massilia plicata*
sus__Acid_0351	Acid_0351	Acidobacteria	*Candidatus Solibacter usitatus*
avm__JQX13_00555	JQX13_00555	Deltaproteobacteria	*Archangium violaceum*
gba__J421_1609	J421_1609	Gemmatimonadetes	*Gemmatirosa kalamazoonesis*
scu__SCE1572_30285	SCE1572_30285	Deltaproteobacteria	*Sorangium cellulosum* So0157-2
scu__SCE1572_38590	SCE1572_38590	Deltaproteobacteria	*Sorangium cellulosum* So0157-2
tsa__AciPR4_0422	AciPR4_0422	Acidobacteria	*Terriglobus saanensis*
scl__sce3052	sce3052	Deltaproteobacteria	*Sorangium cellulosum* So ce56
samy__DB32_000987	DB32_000987	Deltaproteobacteria	*Sandaracinus amylolyticus*
gog__C1280_20305	C1280_20305	Planctomycetes	*Gemmata obscuriglobus*
abas__ACPOL_1890	ACPOL_1890	Acidobacteria	*Acidisarcina polymorpha*
abac__LuPra_05068	LuPra_05068	Acidobacteria	*Luteitalea pratensis*
nmv__NITMOv2_2828	NITMOv2_2828	Nitrospirae	*Nitrospira moscoviensis*
dge__Dgeo_2483	Dgeo_2483	Deinococcus-Thermus	*Deinococcus geothermalis*
dpd__Deipe_0905	Deipe_0905	Deinococcus-Thermus	*Deinococcus peraridilitoris*
ddr__Deide_13120	Deide_13120	Deinococcus-Thermus	*Deinococcus deserti*

dmr__Deima_2064	Deima_2064	Deinococcus-Thermus	*Deinococcus maricopensis*
dfc__DFI_15425	DFI_15425	Deinococcus-Thermus	*Deinococcus ficus*
eco__b0344	b0344	Gammaproteobacteria	*Escherichia coli* K-12 MG1655

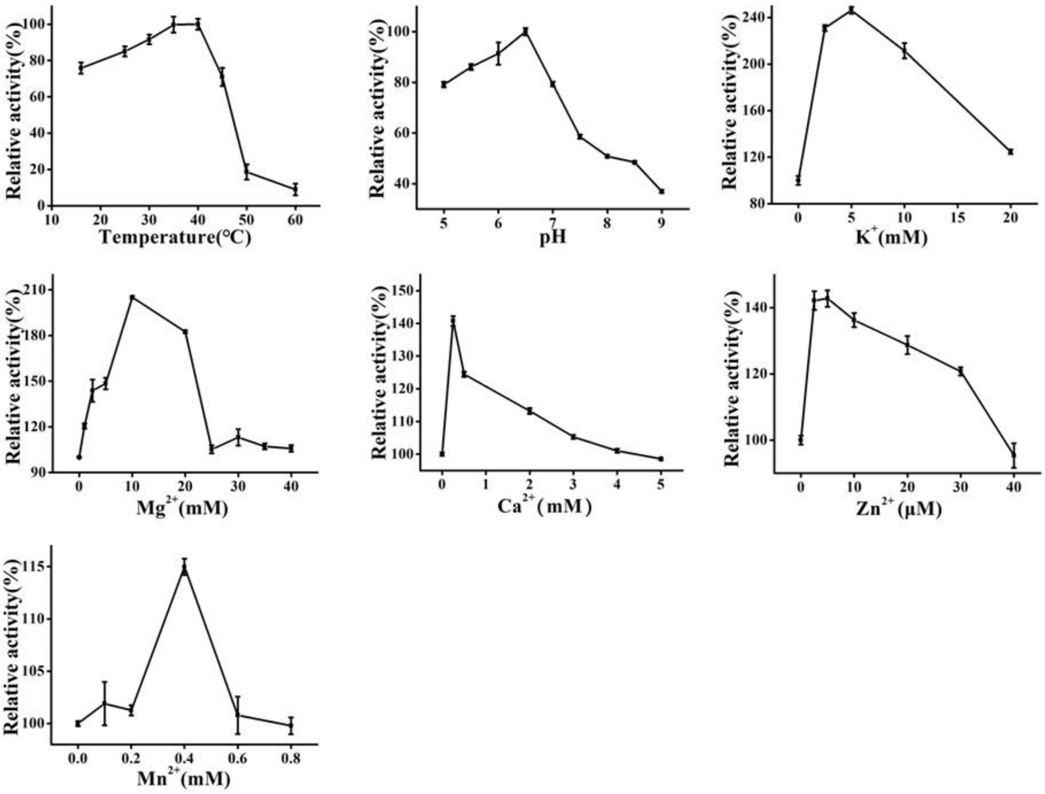

Figure S1 The effects of temperature, pH and metal ions on the enzymatic activity of Nf-LacZ. Relative activities were shown. Data shown are the means ± SD (n = 4). Various concentrations of metal ions (KCl, $MgCl_2$, $CaCl_2$, $ZnCl_2$ and $MnCl_2$) were prepared in 1 ml of 20 mM Tris-HCl (pH 7.0), and the reactions were conducted at 45°C for 1 h with 3 mM ONPG as the substrate. Nf-LacZ concentration, 10 μg/ml.

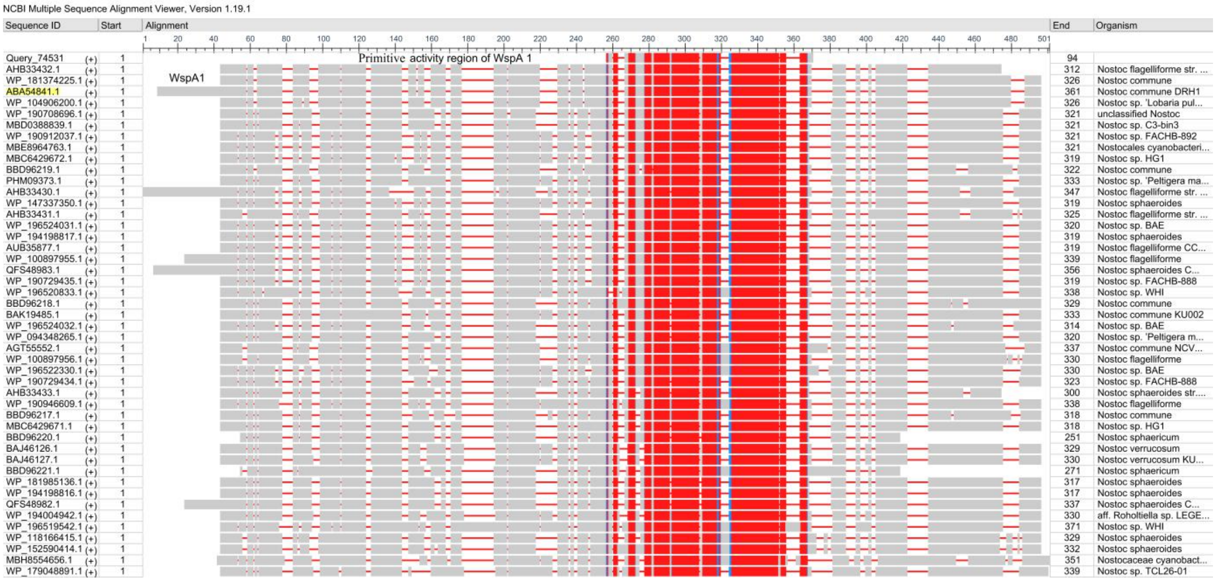

Figure S2 Protein blast against NCBI database using the primitive activity region of WspA1. Query_74531, the primitive activity region of WspA1 (Wsp_{C3}; 94 aa).

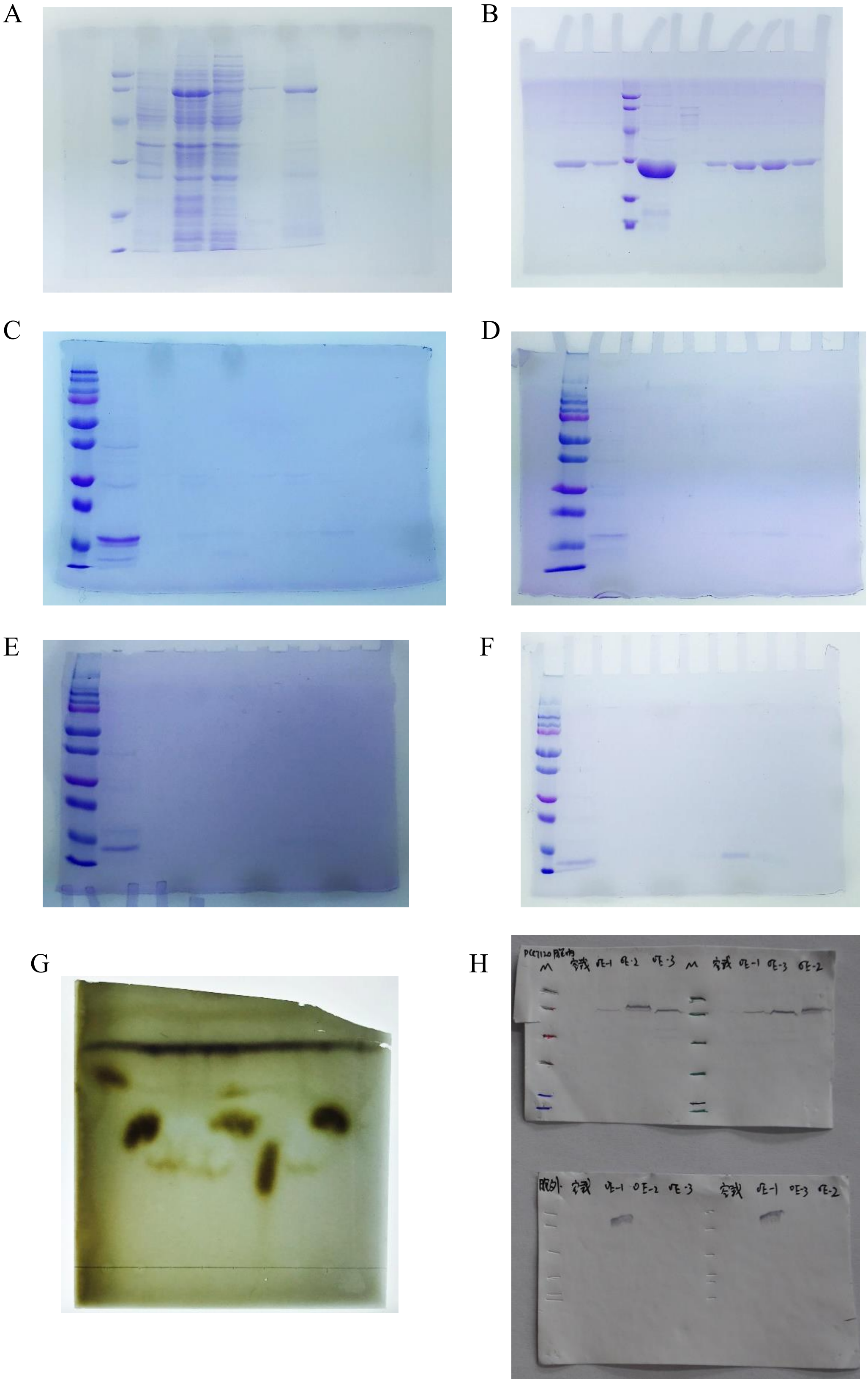

Supplemental figures: uncropped gel/blot images for Fig. 2A (A), Fig. 3A (B), Fig. 5B (C, D, E, and F), Fig. 5E (G), and Fig. 6D (H) in the main article.
